# Pharmacokinetic assessment of vancomycin in critically ill patients and nephrotoxicity prediction using individualized pharmacokinetic parameters

**DOI:** 10.3389/fphar.2022.912202

**Published:** 2022-08-24

**Authors:** Parisa Ghasemiyeh, Afsaneh Vazin, Farid Zand, Elham Haem, Iman Karimzadeh, Amir Azadi, Mansoor Masjedi, Golnar Sabetian, Reza Nikandish, Soliman Mohammadi-Samani

**Affiliations:** ^1^ Department of Clinical Pharmacy, Faculty of Pharmacy, Shiraz University of Medical Sciences, Shiraz, Iran; ^2^ Department of Pharmaceutics, Faculty of Pharmacy, Shiraz University of Medical Sciences, Shiraz, Iran; ^3^ Anesthesiology and Critical Care Research Center, Nemazee Hospital, Shiraz University of Medical Sciences, Shiraz, Iran; ^4^ Department of Biostatistics, School of Medicine, Shiraz University of Medical Sciences, Shiraz, Iran; ^5^ Department of Anesthesiology, Faculty of Medicine, Shiraz University of Medical Science, Shiraz, Iran; ^6^ Trauma Research Center, Shahid Rajaee (Emtiaz) Trauma Hospital, Shiraz University of Medical Sciences, Shiraz, Iran; ^7^ Pharmaceutical Sciences Research Center, Faculty of Pharmacy, Shiraz University of Medical Sciences, Shiraz, Iran

**Keywords:** vancomycin, therapeutic drug monitoring (TDM), pharmacokinetic parameters, critically ill patients, nephrotoxicity, cut-off point, ROC curves, AUC of intervals (AUCτ)

## Abstract

**Introduction:** Therapeutic drug monitoring (TDM) and pharmacokinetic assessments of vancomycin would be essential to avoid vancomycin-associated nephrotoxicity and obtain optimal therapeutic and clinical responses. Different pharmacokinetic parameters, including trough concentration and area under the curve (AUC), have been proposed to assess the safety and efficacy of vancomycin administration.

**Methods:** Critically ill patients receiving vancomycin at Nemazee Hospital were included in this prospective study. Four blood samples at various time intervals were taken from each participated patient. Vancomycin was extracted from plasma samples and analyzed using a validated HPLC method.

**Results:** Fifty-three critically ill patients with a total of 212 blood samples from June 2019 to June 2021 were included in this study. There was a significant correlation between baseline GFR, baseline serum creatinine, trough and peak concentrations, AUCτ, AUC_24h_, Cl, and V_d_ values with vancomycin-induced AKI. Based on trough concentration values, 66% of patients were under-dosed (trough concentration <15 μg/ml) and 18.9% were over-dosed (trough concentration ≥20 μg/ml). Also, based on AUC_24h_ values, about 52.2% were under-dosed (AUC_24h_ < 400 μg h/ml), and 21.7% were over-dosed (AUC_24h_ > 600 μg h/ml) that emphasizes on the superiority of AUC-based monitoring approach for TDM purposes to avoid nephrotoxicity occurrence.

**Conclusion:** The AUC-based monitoring approach would be superior in terms of nephrotoxicity prediction. Also, to avoid vancomycin-induced AKI, trough concentration and AUCτ values should be maintained below the cut-off points.

## 1 Introduction

Vancomycin is a glycopeptide antibiotic considered a drug of choice for targeted or empiric therapy of many Gram-positive bacterial infections (Vazin et al., 2012), especially Methicillin-resistant *Staphylococcus aureus* (MRSA) and Methicillin-resistant coagulase-negative staphylococci (MRCoNS) (Vazin et al., 2018). Vancomycin is a hydrophilic drug with a log P of section 3.1 (Drugbank, 2022). It has an average molecular weight of 1,450 Da (Drugbank, 2022). Since vancomycin has limited oral bioavailability, the main route of administration is intravenous infusion (Estes and Derendorf, 2010). Due to the hydrophilic nature, almost complete urinary excretion would be expectable after intravenous administration. It has been reported that 24 h after single-dose administration of vancomycin, about 80–90% of the drug was recovered unchanged in urine samples (Rybak, 2006). Vancomycin has a narrow therapeutic index that emphasized the necessity of therapeutic drug monitoring (TDM) (Javorska et al., 2016). The most significant adverse reactions related to vancomycin are nephrotoxicity and ototoxicity that are related to the trough (C_min_) and peak (C_max_) concentration values, respectively (Sacristan and Soto, 1992). Risk scoring systems can be recruited to predict vancomycin-associated nephrotoxicity (Kim et al., 2022). Recent guideline regarding the TDM of vancomycin in severe MRSA infections has been reported that trough-only monitoring approach with a target trough concentration of 15–20 μg/ml is no longer recommended in patients with severe MRSA infection due to the lack of efficacy and higher nephrotoxicity occurrence (Rybak et al., 2020). In contrast, it has been reported that the area under the curve (AUC) to minimum inhibitory (MIC) ratio (AUC/MIC) with a target range of 400–600 μg h/ml (assuming MIC values are ≤1 μg/ml) would be an optimal pharmacokinetic/pharmacodynamic (PK/PD) parameter for vancomycin TDM to achieve both adequate clinical efficacy and safety during vancomycin administration (Rybak et al., 2020). The suggested vancomycin dosage in patients with severe MRSA infection is a loading dose of 20–35 mg/kg and a maintenance dose of 15–20 mg/kg (based on actual body weight) every 8–12 h based on TDM data (Ghasemiyeh et al., 2021a). These doses would not be appropriate for target therapeutic AUC/MIC values when MIC is 2 μg/ml (Rybak et al., 2020). In this regard, a 24-h AUC (AUC_24h_) calculation regardless of MIC values would be a suitable alternative to AUC/MIC ratio in these cases. It has been reported that an AUC_24h_ target limit of 700 μg h/ml would be a more reliable pharmacokinetic parameter to achieve sufficient clinical efficacy and prevent vancomycin nephrotoxicity (Zasowski et al., 2018). Critically ill patients admitted to intensive care units (ICU) may have altered pharmacokinetic parameters compared to non-critically ill patients. So, individualized dose adjustment and TDM of vancomycin would be essential in this group of patients to achieve a targeted therapeutic response (Ghasemiyeh et al., 2021b). Also, the results of a recent study revealed that the trough-only monitoring approach should not be considered as a suitable surrogate for AUC_24h_ monitoring in critically ill patients (Turner et al., 2018a). In this study, vancomycin TDM in critically ill patients admitted to the ICUs of Nemazee hospital in Shiraz was assessed. Different pharmacokinetic parameters including trough concentration (C_min_), peak concentration (C_max_), AUC_24h_, AUC of intervals (AUCτ), the volume of distribution (V_d_), clearance (Cl), elimination constant (k), and half-life (t ½) were calculated individually for each patient. Finally, a comparison of the sensitivity of different pharmacokinetic parameters in the prediction of vancomycin-associated nephrotoxicity has been considered. Finally, the ROC curve has been recruited to determine the cut-off points for trough concentration, AUCτ, AUC_24h_, and k to avoid vancomycin-induced AKI occurrence.

## 2 Materials and methods

### 2.1 Materials and equipment

The high-performance liquid chromatography (HPLC) method was used in sample analysis (Azura, Knauer, Germany). HPLC column was C18 (250 mm length × 4.6 mm I.D.; 5 μm pore size), Knauer, Germany. Acetonitrile and methanol were HPLC grade from Merck, Germany, and purchased from a domestic supplier. Ortho-phosphoric acid and sodium hydroxide were from Merck, Germany. Vancomycin standard powder was kindly gifted by Dena Pharmaceutical Company, Tabriz, Iran. Theophylline standard powder was kindly gifted from Exir Pharmaceutical Company, Borujerd, Iran.

### 2.2 Study design and patient population

This is a part of prospective, interventional clinical study which was enrolled between June 2019 and June 2021. A total of 100 patients receiving vancomycin at the ICU ward of Nemazee Hospital, affiliated to Shiraz University of Medical Sciences, Shiraz, Iran, were screened primarily.

The inclusion criteria were critically ill adult patients aged ≥18 years old who were admitted to the ICU of Nemazee Hospital and received vancomycin as an empiric or definite antibiotic regimen. Also, these patients should have a GFR value of ≥45 ml/min (calculated through Cockcroft-Gault equation). Exclusion criteria were age <18 years old, pregnancy, patients with baseline renal failure [including patients with AKI, chronic kidney disease (CKD) stage IIIB or greater, end-stage renal disease (ESRD) patients on hemodialysis], burn injuries, severe hepatic failure (Child-Pugh class C), serum bilirubin level >2.5 mg/dl (Srisawasdi et al., 2010), serum albumin level <2 g/dl, and those receiving theophylline/aminophylline.

### 2.3 Ethical considerations

This study was approved by the Ethics Committee of the Shiraz University of Medical Sciences [Approval ID: IR. SUMS.REC.1398.605] and each patient or their family members are signed written consent forms.

### 2.4 Sampling

Baseline serum creatinine levels were assessed for each patient before vancomycin administration. Also, serum creatinine and creatinine clearance was assessed daily for each participant during vancomycin administration until hospital discharge and/or death occur. Four blood samples were collected from each patient after 48 h of vancomycin initiation to determine steady-state plasma concentrations. Blood samples were collected from these critically ill patients just before the fifth dose administration (trough 1), after the end of 1-h infusion of the fifth dose (peak), 6 h after the fifth dose administration (intermediate), and 12 h after the fifth dose or just before the sixth dose administration (trough 2). AUCτ was calculated in each patient using the trapezoidal method as described in our previous study (Ghasemiyeh et al., 2020).

#### 2.4.1 Sample preparation and HPLC analysis

Five ml sample was taken at each mentioned times (0, 1, 6, and 12 h) and poured into the K_2_EDTA tubes to avoid clot formation. Then, blood samples were centrifuged at 4,000 rpm for 3 min to separate plasma from the whole blood. The 950 µL of the plasma was mixed with 50 µL of theophylline standard solution (with a concentration of 8 mg/L), and samples were vortexed at 2,000 rpm for 1 min. After that, the sample was mixed with 1,000 µL of methanol to precipitate plasma proteins and extract vancomycin from plasma samples. Finally, the mixture was centrifuged at 12,000 rpm for 15 min, and the supernatant was analyzed through a validated HPLC method described in detail before (Usman et al., 2016; Ghasemiyeh et al., 2020). In this regard, the HPLC (Azura, Knauer, Germany) and C18 column (250 mm length × 4.6 mm I.D.; 5 μm pore size; Knauer, Germany) were used for sample analysis. The mobile phase was phosphate buffer (pH of 2.2, 0.03 M) and acetonitrile (86:14 %v/v ratio). The flow rate was 0.72 ml/min and the system was isocratic. The λ_max_ was set at 205 nm and the column temperature was fixed at 25°C (Ghasemiyeh et al., 2020).

### 2.5 Pharmacokinetic assessments

Pharmacokinetic parameters were assessed individually for each participated patient according to Eqs 1–4.

Systemic clearance of the vancomycin was calculated using Eq. 1.

$$Cl=\frac{X_{0}}{\text{AUCτ }}$$ (1)

Where  Cl is clearance in L/h, $X_{0}\;$ is the administered dose in mg in each dosing interval, and AUCτ is the AUC of intervals in mg.h/L. AUCτ was calculated through the trapezoidal method using four blood samples that were taken from each patient. In this study, in order to provide an AUC_24h_-guided vancomycin dosing as reported in recent guidelines (He et al., 2020), we calculate AUC_24h_ for these critically ill patients through the doubling of the AUCτ which was equal to AUC_12h_ in our center. This doubling was conceptualized due to the none significant differences between the first and second trough concentrations in vancomycin receiving patients which emphasis on no drug accumulation possibility during the assessments.

Volume of drug distribution within the body was assessed using Eq. 2.

$$V_{d}=\frac{X_{0}}{C_{max}-C_{min}}$$ (2)

Where $V_{d}\;$ is the volume of distribution in L, $X_{0}\;$ is the administered dose in mg in each dosing interval, $C_{max}$ is peak concentration in mg/L, and $C_{min}$ is trough concentration in mg/L.

Elimination constant of vancomycin was estimated according to Eq. 3.

$$k=\frac{Cl}{V_{d}}$$ (3)

Where k is elimination constant in h^−1^,  Cl is clearance in L/h, and $V_{d}\;$ is the volume of distribution in L.

Elimination half-life of vancomycin was calculated using Eq.4.

$$t_{1/2}=\frac{0.693}{k}$$ (4)

Where $t_{1/2}$ is vancomycin half-life in h and k is elimination constant in h^−1^.

After blood sample analysis, dose adjustment was accomplished according to Eqs 5, 6.

$$k_{0}=\frac{\left(C_{\text{min}}\right)\left(V_{d}\right)\left(k\right)}{\left(1-e^{-k\text{τ}}\right)}$$ (5)

Where $k_{0}$ is the rate of administration in mg/h, $C_{\text{min}}$ is the targeted trough concentration mg/L, $V_{d}\;$ is the volume of distribution in L, k is elimination constant in h^−1^, and τ is the dosing interval in h. (Assuming one-compartment distribution model).

$$\frac{AUC_{2}}{AUC_{1}}=\frac{Dose_{2}}{Dose_{1}}$$ (6)

Where $AUC_{2}$ is the target AUC, $AUC_{1}$ is the current AUC, $Dose_{2}$ is the new required dose to obtain target AUC, and $Dose_{1}$ is the current dose. (Assuming linear pharmacokinetic).

### 2.6 Statistical analysis

The Kolmogorov-Smirnov test was performed to assess the normality of the continuous variable distribution. Continuous variables with normal and abnormal distributions are expressed as mean ± standard deviation (SD) and median with interquartile ranges, respectively. Categorical variables were reported as a percentage. For all statistical analysis, SPSS software (version 25, 2017) was used and *p*-values less than 0.05 were considered to be statistically significant.

#### 2.6.1 Vancomycin-induced AKI

The association between pharmacokinetic parameters, including trough concentration, peak concentration, AUCτ, AUC_24h_, V_d_, Cl, k, t ½ with vancomycin-induced AKI was assessed using the Independent Sample *t*-test and Mann-Whitney test. Also, the association between baseline renal function (including baseline serum creatinine and baseline GFR) and vancomycin-induced AKI were assessed in the same way.

The Pearson correlation test was used to assess the correlation between baseline serum creatinine as well as baseline GFR with each pharmacokinetic parameter.

##### 2.6.1.1 AKI occurrence in different groups of patients regarding their trough and AUC values

A comparison of AKI occurrence in different groups of patients based on trough concentration and AUC values was performed using the Chi-square test. In this regard, patients were divided into four groups based on trough concentration values (trough concentration of <10, 10–15, 15–20, and ≥20 μg/ml). Also, patients were divided into three groups based on AUCτ and AUC_24h_ values (AUCτ <200, 200–300, and ≥300 μg h/ml and AUC_24h_ < 400, 400–600, and ≥600 μg h/ml). Then the occurrence of AKI was compared between these groups using the Chi-square test.

##### 3.6.1.2 Trough concentration values in patients with targeted AUCτ and AUC_24h_ values

In order to evaluate the suitability of using trough-based monitoring approach as a more practical and convenient surrogate for AUC-based monitoring approach, the distribution of trough concentration values in patients who had targeted AUC values of 200–300 μg h/ml and 400–600 μg h/ml for AUCτ and AUC_24h_ respectively were assessed.

##### 2.6.1.3 ROC curve and cut-off point calculation for AKI occurrence

The receiver operating characteristic (ROC) curve was exploited to determine the cut-off point of AKI occurrence regarding trough concentration, AUCτ, and AUC_24h_ values (Han et al., 2014).

## 3 Results

### 3.1 Patient population

**FIGURE 1:**
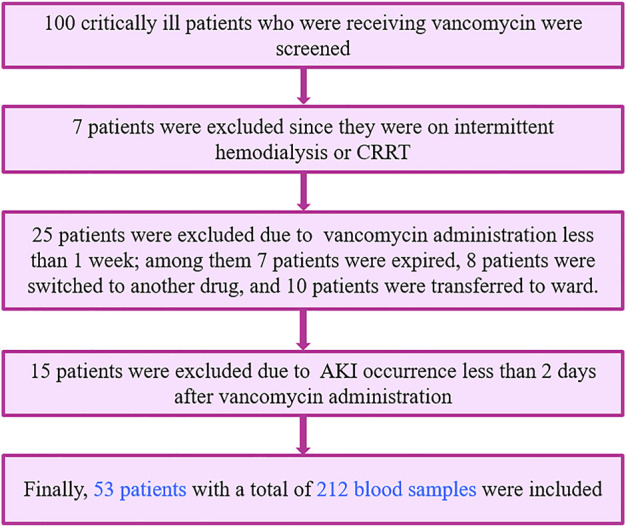
Patients’ screening and selection based on inclusion and exclusion criteria.

A total of 100 critically ill patients were screened. 53 patients, with a total of 212 blood samples, were included based on the inclusion and exclusion criteria and completed this study as shown in Figure 1. Participated patients had almost equal distribution regarding their gender, 52.8% of them were male, and the remaining were female. These critically ill patients were between 18 and 85 years old with an average of 50.25 ± 19.12 years. Among these patients, 28.3% received vancomycin as definite therapy with confirmed positive blood culture (especially MRCoNS and MRSA), and the remaining (71.7%) received vancomycin as an empiric therapy regimen. Among these included critically ill patients, 43.4% of them were septic. Demographic characteristics, clinical, and laboratory data of the included patients have been shown in Table 1.

**TABLE 1:** Patients’ demographic characteristics and laboratory data.

Characteristics	Values
Total number of patients	53 (212 blood samples)
Gender (Male/Female)	28/25 (52.8%/47.2%)
Type of administration (Empiric/Definite)	38/15 (28.3%/71.7%)
Type of infection	
Pneumonia	24 (45.29%)
Meningitis	8 (15.09%)
Other CNS infections	15 (28.30%)
Skin and soft tissue infection	5 (9.43%)
Intra-abdominal infection	1 (1.89%)
Age (years old)	18–85 (Mean ± SD^a^: 50.25 ± 19.12)
APACHE II^b^ score	4–32 (Mean ± SD: 16.86 ± 7.32)
IBW^c^ (kg)	44–95 (Mean ± SD: 65.52 ± 10.16)
Height (cm)	140–185 (Mean ± SD: 166.62 ± 9.72)
BMI^d^	18–33.56 (Mean ± SD: 26.08 ± 3.75)
Baseline serum creatinine (mg/dl)	0.5–2.2 (Mean ± SD: 0.95 ± 0.32)
Baseline creatinine clearance (GFR^e^) (ml/min)	46.03–146.23 (Mean ± SD: 91.70 ± 25.68)
Conjugated bilirubin (mg/dl)	0.10–0.90 (Mean ± SD: 0.30 ± 0.20)
Total bilirubin (mg/dl)	0.20–2.5 (Mean ± SD: 0.80 ± 0.58)
Serum albumin (g/dl)	2.5–4.7 (Mean ± SD: 3.37 ± 0.56)

a Acute physiology and chronic health evaluation II (APACHE II) is a severity-of-disease classification system that is used in critically ill patients admitted to ICU, to estimate the ICU, mortality.

b Ideal body weight.

c Body mass index.

d Glomerular filtration rate (using Cockcroft-Gault equation).

e Standard deviation.

### 3.2 Drug administration and sample analysis

**FIGURE 2:**
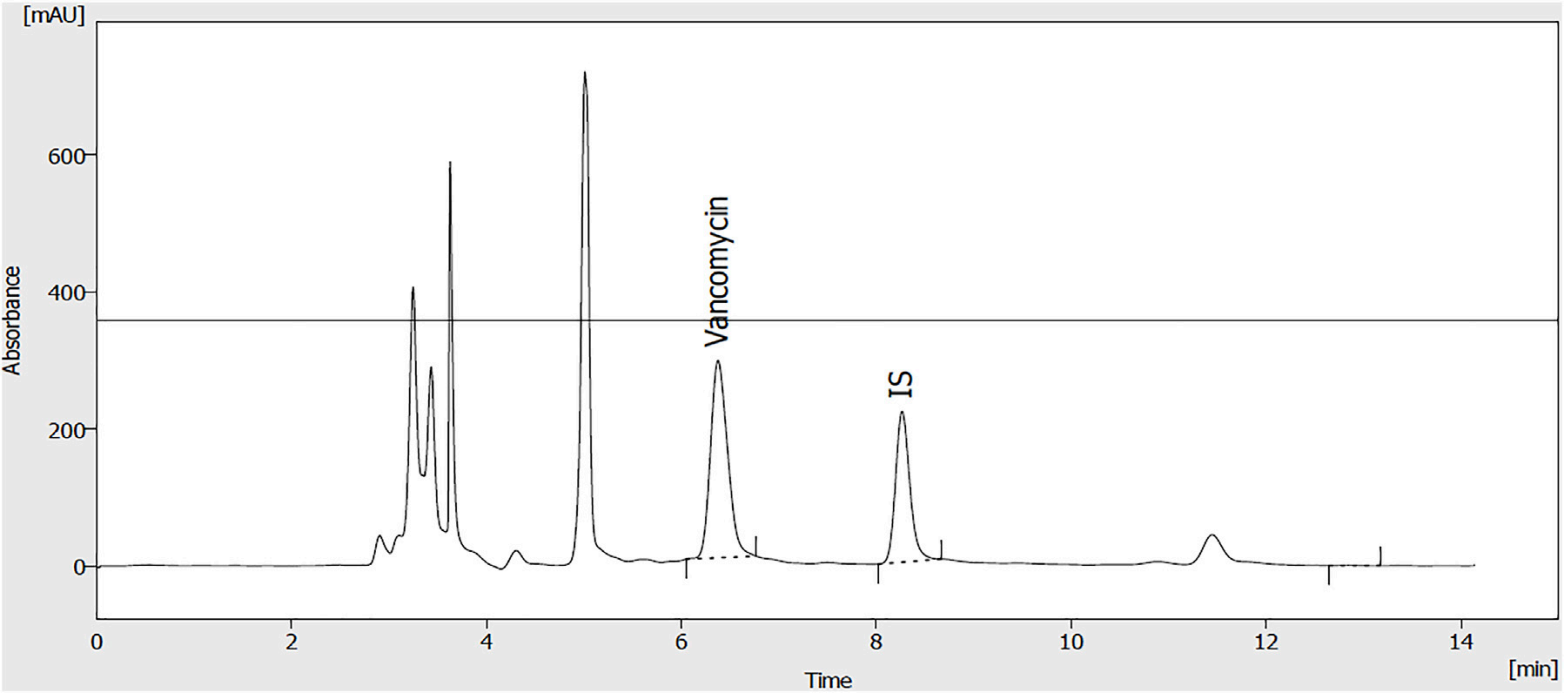
A representative chromatogram of vancomycin (50 μg/ml) and internal standard (theophylline; 20 μg/ml) extracted from plasma samples.

Vancomycin was administered with a loading dose of 20–35 mg/kg and a maintenance dose of 15–20 mg/kg Q8-12 h. Then doses were adjusted based on pharmacokinetic parameters individually, as mentioned in Eqs 5, 6. A representative chromatogram of vancomycin analysis using the HPLC method has been presented in Figure 2. As shown in Figure 2, vancomycin and theophylline (as an internal standard) had retention times of 6.7 and 8.2 min, respectively.

### 3.3 Pharmacokinetic assessments

According to the four samples (0, 1, 6, and 12 h) taken from each patient, different pharmacokinetic parameters including V_d_, Cl, k, t ½, AUCτ, and AUC_24h_ were assessed individually and summarized in Table 2.

**TABLE 2:** Pharmacokinetic parameters after vancomycin administration at steady-state condition.

Pharmacokinetic parameters	Minimum-maximum range (Mean ± SD)
Trough concentration 1 (C_ss_^min^) (µg/ml)	0–69.36 (14.51 ± 12.90)
Peak concentration (1h) (C_ss_^max^) (µg/ml)	5.27–122.30 (30.35 ± 22.54)
Intermediate concentration (6h) (µg/ml)	2.99–76.60 (19.95 ± 17.34)
Trough concentration 2 (C_ss_^min^) (µg/ml)	0–69.36 (14.43 ± 13.65)
AUCτ^1^ (µg.h/ml)	47.57–1030.96 (248.79 ± 207.08)
AUC_24h_ (µg.h/ml)	95.14–2061.92 (497.58 ± 414.16)
V_d_ (L)	18.89–689.66 (133.14 ± 22.42)
Cl (L/h)	0.97–21.02 (6.92 ± 5.27)
k (h^−1^)	0.01–0.28 (0.076 ± 0.057)
t ½ (h)	2.48–63.78 (14.94 ± 12.05)

### 3.4 Vancomycin-induced AKI

Results of this study revealed that 17% of the present study population experienced AKI during vancomycin administration. 24.5% of the participated patients received at least one nephrotoxic agent other than vancomycin. The effect of concomitant other nephrotoxic medications administration was omitted using multivariate logistic regression. None of these included patients were co-treated with piperacillin-tazobactam. According to the latest recommendation of Uptodate®, the combination therapy with vancomycin and piperacillin-tazobactam is no longer supported and it should be avoided due to the higher incidence of acute kidney injury. Therefore, this combination is no longer used in our medical center. The association between each pharmacokinetic parameter value and vancomycin-induced AKI has been summarized in Table 3. There was a significant association between each of the trough concentration, peak concentration, AUCτ, AUC_24h_, V_d_, and Cl and vancomycin-induced AKI. Furthermore, these results revealed that there was a significant association between baseline serum creatinine and baseline GFR with vancomycin-induced AKI.

**TABLE 3:** The association between pharmacokinetic parameters of vancomycin and vancomycin-induced AKI.

Pharmacokinetic	*p*-value
Baseline serum creatinine	0.024
Baseline GFR^a^	0.009
Trough concentration 1 (0h)	0.020
Peak concentration (1h)	0.020
Trough concentration 2 (12h)	0.017
AUCτ	0.015
AUC_24h_	0.015
V_d_	0.032
Cl	0.028
k	0.138
t ½	0.259

a Glomerular filtration rate.

### 3.5 Correlation between baseline renal function and pharmacokinetic parameters

The correlation between baseline serum creatinine and baseline GFR as indicators of baseline renal function and pharmacokinetic parameters of vancomycin are summarized in Table 4. There was a significant correlation between each of trough concentration 2 (12 h) and t ½ with baseline serum creatinine. Also, there was a significant correlation between each of trough concentration, peak concentration, AUCτ, and AUC_24h_ and baseline GFR.

**TABLE 4:** The correlation between baseline serum creatinine and baseline GFR and pharmacokinetic parameters.

Pharmacokinetic	*p*-value	Pearson correlation coefficient
Baseline serum creatinine		
Baseline GFR^a^	0.000	−0.608
Trough concentration 1 (0h)	0.057	0.263
Peak concentration (1h)	0.224	0.183
Trough concentration 2 (12h)	0.048	0.273
AUCτ	0.140	0.221
AUC_24h_	0.140	0.221
V_d_	0.888	0.021
Cl	0.237	−0.178
k	0.313	−0.152
t ½	0.046	0.296
Baseline GFR^a^		
Baseline serum creatinine	0.000	−0.608
Trough concentration 1 (0h)	0.016	−0.330
Peak concentration (1h)	0.031	−0.318
Trough concentration 2 (12h)	0.020	−0.318
AUCτ	0.032	−0.317
AUC_24h_	0.032	−0.317
V_d_	0.064	0.275
Cl	0.088	0.254
k	0.580	0.084
t ½	0.632	0.073

a Glomerular filtration rate.

### 3.6 AKI occurrence in different groups of patients regarding their trough and AUC values

The frequency and percentage of critically ill patients in different groups based on their trough concentrations and AUC values have been shown in Table 5. Also, comparison of AKI occurrence in different groups of patients based on trough concentration and AUC values has been summarized in Table 5. There was a significant association between each of trough 1 (0 h), trough 2 (12 h), AUCτ, and AUC_24h_ with vancomycin nephrotoxicity.

**TABLE 5:** Comparison of occurrence of vancomycin-induced AKI in different groups of patients based on trough concentration and AUC values using Chi-square test (*N* = 53).

Pharmacokinetic parameter	Groups	Frequencies (%)	*p*-value
Trough concentration 1 (µg/ml)	<10	37.70	<0.001
	10–15	28.30	
	15–20	15.10	
	≥20	18.90	
Trough concentration 2 (µg/ml)	<10	41.50	<0.001
	10–15	26.40	
	15–20	15.10	
	≥20	17.00	
AUCτ (µg.h/ml)	<200	52.20	0.001
	200–300	26.10	
	≥300	21.70	
AUC_24h_ (µg.h/ml)	<400	52.20	0.001
	400–600	26.10	
	≥600	21.70	

### 3.7 Trough concentration values in patients with targeted AUCτ and AUC_24h_ values

Trough concentration values in patients with targeted AUC values, including AUCτ of 200–300 μg h/ml, and AUC24 h of 400–600 μg h/ml have been summarized in Table 6.

**TABLE 6:** Trough levels in patients with targeted AUC values (*N* = 12).

Targeted AUC values	Trough concentration	Categories (µg/ml)	Frequencies (%)
AUCτ = 200–300 μg h/ml	Trough concentration 1 (0 h)	<10	0.00
		10–15	33.33
		15–20	33.33
		≥20	33.33
	Trough concentration 2 (12)	<10	0.00
		10–15	50.00
		15–20	25.00
		≥20	25.00
AUC_24h_ = 400–600 μg h/ml	Trough concentration 1 (0 h)	<10	0.00
		10–15	33.33
		15–20	33.33
		≥20	33.33
	Trough concentration 2 (12)	<10	0.00
		10–15	50.00
		15–20	25.00
		≥20	25.00

### 3.8 ROC curve and cut-off point calculation for AKI occurrence

**FIGURE 3:**
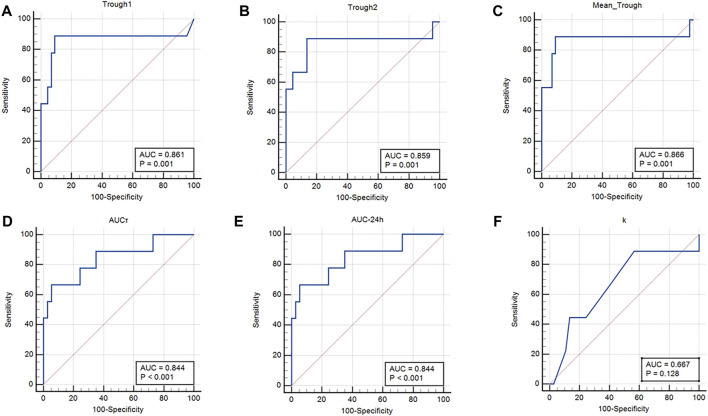
Area under the ROC curves. **(A)** Trough concentration 1 (0 h); **(B)** Trough concentration 2 (12 h); **(C)** Mean trough concentration; **(D)** AUCτ, **(E)** AUC_24h_; and **(F)** Elimination constant K.

Results of the ROC curve regarding cut-off point determination for AKI occurrence have been summarized in Table 7. Also, the area under the ROC curves that indicate the sensitivity and specificity of each parameter in the prediction of vancomycin-induced AKI (Figure 3; Table 7).

**TABLE 7:** Trough concentration and AUC cut-off points for AKI occurrence and the reported area under the ROC curve values.

Pharmacokinetic parameter	Cut-off point for AKI	Area under the ROC curve (AUC)	Sensitivity (%)	Specificity (%)
Trough concentration 1	16.78 μg/ml	0.861	88.9	90.9
Trough concentration 2	15.86 μg/ml	0.859	88.9	86.4
Mean trough concentration	16.92 μg/ml	0.866	88.9	90.9
AUCτ	355.19 μg h/ml	0.844	66.7	94.6
AUC_24h_	710.38 μg h/ml	0.844	66.7	94.6
k	0.06 h^−1^	0.667	88.9	43.2

## 4 Discussion

Most of the published papers on vancomycin pharmacokinetic and TDM studies are retrospective and observational (Póvoa et al., 2021). In this study, we designed a prospective interventional study of vancomycin pharmacokinetic assessments in critically ill patients and dose adjustments were done for all participants based on Eqs 5, 6 and individualized pharmacokinetic parameters. Results of this study revealed that there was a significant correlation between baseline GFR, baseline serum creatinine, trough and peak concentrations, AUCτ, AUC_24h_, Cl, and V_d_ values with vancomycin-induced AKI. The cut-off points for vancomycin-induced AKI were 16.92 μg/ml, 355.19 μg h/ml, and 710.38 μg h/ml for trough concentration, AUCτ, and AUC_24h_, respectively.

### 4.1 Pharmacokinetic assessments

Results of the pharmacokinetic analysis revealed that critically ill patients had average steady-state trough concentrations of 14.51 ± 12.90 and 14.43 ± 13.65 μg/ml at 0 and 12 h (for a 12-h interval dosing), respectively. The approximately same trough values indicated that steady-state conditions had been achieved during sample preparation. The mean values of AUCτ and AUC_24h_ were 248.79 ± 207.08 and 497.58 ± 414.16 μg h/ml, respectively. These were lower than the results of the recent pharmacokinetic study in critically ill patients that reported the mean ± SD AUC_24h_ of 604 ± 109 μg h/ml using the linear-log trapezoidal rule for AUC calculation (Turner et al., 2018b). Also, results of the another pharmacokinetic study on critically ill patients was receiving vancomycin reported mean ± SD AUC_24h_ of 665.9 ± 136.5 μg h/ml which was higher than the results of present study and can be attributed to the heterogeneity of these critically ill patients with multiple complications (Shahrami et al., 2016). Results of a retrospective study confirmed that vancomycin AUC/MIC values of ≥400 in patients with entrococcal infection was significantly superior to AUC/MIC values of <400 in terms of clinical response and microbiological response. However, the rate of vancomycin induced-AKI was significantly higher in the former group (Katip and Oberdorfer, 2021). The wide range of pharmacokinetic parameters in these patients confirmed the altered pharmacokinetic characteristics in critically ill patients due to various physiologic and pathologic changes in patients conditions (Boucher et al., 2006; Ghasemiyeh et al., 2021a). Sepsis, a common cause of death in critically ill patients, can result in many physiologic changes, including capillary leak syndrome, cytokine release syndrome, vasodilation enhancement, and altered protein biosynthesis. These physiologic changes in septic patients can induce altered pharmacokinetic parameters (Radke et al., 2017). Also, altered plasma protein binding in critically ill patients can result in a higher volume of distribution (V_d_) values that emphasize the necessity of higher dose requirements of hydrophilic drugs such as vancomycin (Zeind and Carvalho, 2018). Another altered pharmacokinetic parameter in critically ill patients would be creatinine clearance enhancement that would be due to the augmented renal clearance (ARC) phenomenon and acute kidney injury (AKI) occurrence (Zeind and Carvalho, 2018). The ARC in critically ill patients was accompanied with higher Cl values, shorter drug half-lives, and lower AUC values. Therefore, the required vancomycin dose to obtain optimum clinical response and reach to the targeted trough concentration and AUC values was enhanced accordingly (Sridharan et al., 2019). Also, augmented V_d_ and Cl_V_ values in septic critically ill patients emphasize the necessity of close therapeutic monitoring of vancomycin and higher dose administration, respectively (Roberts and Lipman, 2006). The enhanced V_d_ values of hydrophilic drugs including vancomycin, both in AKI and critically ill patients, could be attributed to the volume overload and decreased vancomycin protein binding [enhanced the fraction of unbound drug (Fu)]. Therefore, administration of higher loading dose amounts would be required (Eyler and Mueller, 2011). In addition, in patients with vancomycin-induced AKI, although the Cl_V_ and vancomycin renal elimination have been diminished, however, the residual kidney function along with the possible renal replacement therapies can enhance the net Cl_V_ values that should be considered in maintenance dose adjustment (Eyler and Mueller, 2011). Since vancomycin-induced AKI may be irreversible in many critically ill patients, vancomycin TDM using optimal pharmacokinetic parameters is essential to prevent morbidities and mortalities related to AKI occurrence in critically ill patients (Rybak et al., 2020). These results emphasized the necessity of the estimation of the individualized pharmacokinetic parameters (including V_d_, Cl, k, and t ½) for each patient.

### 4.2 Vancomycin-induced AKI

In this study, 17% of the participated critically ill patients developed AKI during vancomycin treatment. The incidence of vancomycin-associated nephrotoxicity in our center was higher than that of a previous retrospective study in critically ill Japanese patients receiving vancomycin with AKI incidence of approximately 12% (13 out of 109 patients) (Chuma et al., 2018). In another study, the rate of vancomycin nephrotoxicity has been reported in range of 5% to 35%, respectively, (Karimzadeh et al., 2017). Results of a retrospective cohort study revealed that the rate of drug-induced AKI in the group of patients who were treated with concomitant vancomycin and piperacillin-tazobactam was significantly higher in comparison to those who were on concomitant vancomycin and cefepime regimen with AKI occurrence rate of 29% and 11%, respectively. Furthermore, the onset of AKI occurrence was significantly more rapid in the former group (Navalkele et al., 2017). Results of another study revealed that the occurrence of vancomycin-induced AKI was significantly higher in those who received concomitant vancomycin and piperacillin-tazobactam regimen in comparison to those who didn’t receive this combination. In addition, the results of this study revealed that the occurrence of vancomycin-induced AKI in both groups was significantly lower in those who received AUC-guided dosing services in comparison to those with trough-guided dosing approach. The rate of AKI incidences in AUC-guided dosing group were 13.6% and 3.8% in concomitant vancomycin and piperacillin-tazobactam group and non-concomitant group, respectively. While in trough-guided dosing group these values were 17.8% and 7.4%, respectively (Muklewicz et al., 2021). The association between baseline renal function and AKI occurrence revealed that baseline serum creatinine and GFR values were significantly associated with AKI occurrence. However, a retrospective study on non-dialysis patients revealed that the baseline renal function had no significant correlation with neither clinical efficacy nor nephrotoxicity occurrence during vancomycin vs. linezolid therapy in patients with MRSA pneumonia (Liu et al., 2017). Also, there was a highly significant association (*p*-value <0.0001) between each trough concentration, peak concentration, AUCτ, AUC_24h_, Cl, V_d_ with AKI occurrence, which indicates that these pharmacokinetic parameters would be considered as suitable parameters in vancomycin-induced AKI prediction. As mentioned in drug monographs including Lexi-Comp®, vancomycin administration schedule would be different between patients, varied from once a day administration to three doses per day; therefore, considering AUCτ, instead of AUC_24h_ would be a more convenient parameter. Higher trough, peak, AUCτ, and AUC_24h_ values, indicated the higher incidence of vancomycin-induced AKI. Results of a recent exposure-toxicity meta-analysis study revealed that the higher initial trough concentration and maximum trough concentration were significantly associated with the higher incidence of vancomycin-induced AKI (Bellos et al., 2020) which was consistent with our study results. Also, the results of a recent meta-analysis study emphasized that the AUC_24h_ values of <650 μg h/ml could significantly reduce the incidence of vancomycin-induced AKI (Aljefri et al., 2019).

Furthermore, results of a recent retrospective study on the Veteran population revealed that the incidence of vancomycin nephrotoxicity in patients with AUC_24h_ values of ≥550 μg h/ml was significantly higher in comparison to those with AUC_24h_ values of <550 μg h/ml (Poston-Blahnik and Moenster, 2021). Lower Cl values showed a higher risk of vancomycin-induced AKI which could be attributed to the more accumulation of drug within the body secondary to reduced Cl. This results in higher cumulative AUC (AUC_CUM_) values which are significantly associated with a higher rate of vancomycin nephrotoxicity (Kloprogge et al., 2019). These findings regarding pharmacokinetic parameters were compatible with previous researches on vancomycin TDM (Pritchard et al., 2010; Finch et al., 2017a). Also, our results revealed that the lower the V_d_ values, the higher the incidence of vancomycin-induced AKI. There was no significant association between both t ½ and k with vancomycin-induced AKI (*p*-value = 0.259 and *p*-value = 0.138, respectively).

Results of the pharmacokinetic parameters assessments in present study revealed a significant association between each trough concentration, peak concentration, AUCτ, and AUC_24h_, Cl, and V_d_ with vancomycin-induced AKI. With higher trough, peak, AUCτ, and AUC_24h_ values, the higher incidence of AKI. In contrast, with lower Cl values, there was higher incidence of vancomycin-induced AKI.

### 4.3 Correlation between baseline renal function and pharmacokinetic parameters

According to our results, there was no significant correlation between trough concentration 1, peak concentration, AUCτ, AUC_24h_, V_d_, Cl, and k, with baseline serum creatinine. In contrast, a significant correlation was seen between trough concentration 1, trough concentration 2, peak concentration, AUCτ, and AUC_24h_ with baseline GFR. These results once more emphasized the superiority of GFR over serum creatinine in predicting renal function status. As a classic marker of renal function, serum creatinine has several limitations in the early detection of renal dysfunction especially in critically ill patients (Sagheb et al., 2014). In present study, there was statistically significant negative correlations between baseline GFR and each of trough concentration, peak concentration, AUCτ, and AUC_24h_. No significant correlation was observed between V_d_, Cl, k, and t½ with baseline GFR. There was a highly significant negative correlation between baseline serum creatinine and baseline GFR, which is completely compatible with the Cockcroft-Gault equation for GFR calculation (Yamaki and Nguyen, 2020). Results of a previous study on septic patients revealed that the GFR (Cl_Cr_) with higher sensitivity and higher ROC area under the curve was superior to serum creatinine in terms of prediction of insufficient vancomycin trough concentration. The sensitivity of GFR and serum creatinine in prediction were 26% and 11%, respectively. While the ROC area under the curve values for GFR and serum creatinine were 0.75 and 0.69, respectively (Ocampos-Martinez et al., 2012). Results of a previous pharmacokinetic study in patients with various stages of kidney disease including GFR>60 ml/min, GFR 10–60 ml/min, and GFR<10 ml/min revealed that there was no significant correlation between V_d_ and baseline GFR that was incompatible with our results. However, a significant negative correlation was seen between mean half-life and baseline GFR along with a positive correlation between Cl and baseline GFR (Matzke et al., 1984). These contradictory results from our study would be attributed to the exclusion of patients with GFR<45 ml/min in our pharmacokinetic study.

### 4.4 AKI occurrence in different groups of patients regarding their trough and AUC values

The rate of vancomycin-induced AKI occurrence was significantly different in groups of patients based on trough concentrations, AUCτ, and AUC_24h_ values. Higher values of these parameters indicate a higher rate of AKI occurrence, which was in compatible with the results of previous pharmacokinetic studies (Finch et al., 2017b; Bellos et al., 2020). In addition, the results of a retrospective study on critically ill patients which categorized patients based on their trough concentration values into 4 groups of trough concentrations of <10, 10–15, 15–20, and ≥20 μg/ml, revealed that the early-onset vancomycin-induced AKI was significantly associated with trough concentrations of ≥20 μg/ml. Also, they reported that the occurrence of vancomycin-induced AKI in the group with a trough concentration of ≥20 μg/ml was significantly higher than that of the group with a trough concentration of <10 μg/ml (31.3% vs. 6.3%) (Chuma et al., 2018).

### 4.5 Trough concentration values in patients with targeted AUCτ and AUC_24h_ values

According to the recently published guidelines, vancomycin TDM should be performed based on AUC_24h_ values of 400–600 μg h/ml. Accordingly, in this study, we suggest the AUCτ values of 200–300 μg h/ml for a 12-h dosing interval regimen to reach optimal efficacy and prevent vancomycin nephrotoxicity. Results of our study revealed that 33.33% (4 out of 12) of critically ill patients with these targeted AUC values (AUC_24h_ of 400–600 μg h/ml and AUCτ of 200–300 μg h/ml) had trough one concentration of 10–15 μg/ml, 33.33% of them had trough one values of 15–20 μg/ml, and the remaining 33.33% had trough one values of ≥20 μg/ml. These percentages were 50% (6 out of 12), 25%, and 25%, for trough two concentrations, respectively. So, these results revealed that up to 33.33–50% of patients with trough concentrations <15 μg/ml could reach the targeted AUC values. These lower trough values could prevent higher drug exposure and further unwanted nephrotoxicity. These results were compatible with the previous study on vancomycin pharmacokinetic which revealed that among patients with AUC_24h_ ≥ 400 μg/ml, 59% had trough concentration <15 μg/ml, 18% had trough concentration of 15–20 μg/ml, and 23% had trough concentration of ≥20 μg/ml (Neely et al., 2014). So, trough concentration would not be considered as a sole suitable surrogate of AUC monitoring (Neely et al., 2014; Lodise and Drusano, 2021; Póvoa et al., 2021; Tsutsuura et al., 2021). However, still trough-based monitoring approach has been considered as the most routine practical method of vancomycin TDM in different center around the world because of the ease of blood sampling and dosing adjustment (Stewart et al., 2021). Results of a prospective study revealed that among all the enrolled patients, 19% of all measured trough concentrations were in therapeutic ranges of 15–20 μg/ml, while 70% of all calculated AUC values had adequate ranges which support the superiority of AUC-guided dosing over the trough only-based vancomycin dosing. In addition, the results of this study revealed that the vancomycin AUC-based dose adjustment was accompanied with reduced the required plasma samples preparation, shorter duration of antibiotic therapy, and reduced the risk of vancomycin-induced AKI in comparison to trough only-based monitoring approach. The clinical efficacy was equal in these monitoring approaches (Neely et al., 2018).

### 4.6 ROC curve and cut-off point calculation for AKI occurrence

Results of the ROC curve revealed that trough concentration, AUCτ, and AUC_24h_ are the best pharmacokinetic parameters for vancomycin-induced AKI prediction. Also, k would be as other pharmacokinetic parameters with lower sensitivity for AKI prediction. The suggested cut-off points in which the higher values would be significantly associated with vancomycin-induced AKI occurrence were 16.92 μg/ml for mean trough concentration with the area under the ROC curve of 0.866. In addition, the suggested cut-off points for AUCτ and AUC_24h_ were 355.19 and 710.38 μg h/ml, respectively with the area under the ROC curve of 0.844. Although the sensitivity of trough concentrations was higher (88.9%), however, the AUCτ and AUC_24h_ had higher specificity values (94.6%) in vancomycin-induced AKI prediction. Results of a previous study on vancomycin pharmacokinetic reported that the cut-off point of 12.1 μg/ml for vancomycin trough concentration should be considered to prevent vancomycin nephrotoxicity (Han et al., 2014). According to another pharmacokinetic study on critically ill patients, vancomycin trough concentration threshold of 16.5 μg/ml is crucial to prevent vancomycin-induced AKI occurrence which was more compatible with the results of the present study (Hanrahan et al., 2015). In another study that was performed on Brazilian critically ill patients, vancomycin trough concentration threshold of 17.53 μg/ml with sensitivity, specificity, and ROC area under the curve of 79.7%, 83.3%, and 0.806, respectively was reported as predictor of vancomycin-induced AKI between the second and fourth days of vancomycin administration (Zamoner et al., 2022). Also, a recent meta-analysis study has reported that the cut-off point of 650 μg h/ml for AUC_24h_ should be maintained to prevent vancomycin-induced AKI. According to this study, using the AUC-monitoring approach could significantly lower the incidence of nephrotoxicity compared to the trough-only-based method (Aljefri et al., 2019).

Furthermore, the suggested cut-off point of 0.06 h^−1^ for k with the area under the ROC curve of 0.667 and sensitivity of 88.9% would be an alternative pharmacokinetic parameter for vancomycin-induced AKI prediction with lower specificity amounts (43.2%). The k values lower than the suggested cut-off point would be associated with vancomycin accumulation and consequently AKI occurrence.

### 4.7 Study limitations

This study is a part of a prospective, interventional study in a single-center that included critically ill patients from the general and central ICUs of Nemazee Hospital, Shiraz, Iran. It has no matched control group that included critically ill patients from the general and central ICU of Nemazee Hospital, Shiraz, Iran. So, further larger clinical trials with the control group are required to confirm these results. In this study, vancomycin was administered with the usual dosage. The results of this study may not reproducible and cannot be extrapolated to the critically ill patients with augmented renal clearance (ARC) phenomenon. So, dose simulation and population pharmacokinetic studies are required to confirm these results. Finally, it would be better to consider the effect of pharmacogenetics and gene polymorphisms on the occurrence of vancomycin nephrotoxicity.

## 5 Conclusion

According to the results of this prospective study and individualized pharmacokinetic parameters, dosing justification is necessary almost in 50% of the patients. Although a significant correlation was seen between each of trough concentration, peak concentration, AUCτ, AUC_24h_, V_d_, Cl, baseline serum creatinine, and baseline GFR values and vancomycin-induced AKI, but AUC-based monitoring approach for vancomycin TDM would be superior to trough-only monitoring approach in terms of nephrotoxicity prediction. Also, in order to avoid vancomycin-induced AKI, trough concentration, AUCτ, and AUC_24h_, values should be maintained below the cut-off points including 16.92 μg/ml, 355.19 μg h/ml, and 710.38 μg h/ml respectively.

The main focus of this study was on calculation and application of AUCτ along with AUC_24h_ to assess vancomycin efficacy and also vancomycin-associated nephrotoxicity in critically ill patients that has not been reported elsewhere. The main advantage of AUCτ would be the reduction in the number of required plasma samples to calculate the time-concentration curve area in comparison to the AUC_24h_, especially in those who are receiving vancomycin with a dosing interval different from the daily dosing schedules (ie., 6, 8, 12, or 48 h). In addition, through the recruitment of AUCτ, faster assessments of various pharmacokinetic parameters, vancomycin dose adjusting, and decision-making about patients’ pharmacotherapy regimens would be obtained. Also, the correlation of GFR and serum creatinine values with pharmacokinetic parameters has been assessed which confirmed the superiority of GFR in terms of nephrotoxicity prediction and correlation with pharmacokinetic parameters. Furthermore, in this study, the cut-off points of AUCτ, AUC_24h_, and trough concentrations have been reported in critically ill patients using ROC curves to avoid vancomycin-associated nephrotoxicity which has not been reported together elsewhere.

## Data Availability

The original contributions presented in the study are included in the article/Supplementary Material, further inquiries can be directed to the corresponding authors.
